# Individual differences in working memory capacity are unrelated to the magnitudes of retrocue benefits

**DOI:** 10.1038/s41598-021-86515-5

**Published:** 2021-03-31

**Authors:** Chaoxiong Ye, Qianru Xu, Xinyang Liu, Piia Astikainen, Yongjie Zhu, Zhonghua Hu, Qiang Liu

**Affiliations:** 1 Institute of Brain and Psychological Sciences, Sichuan Normal University, Chengdu, China; 2 Department of Psychology, University of Jyvaskyla, Jyvaskyla, Finland; 3 Department of Computer Science, University of Helsinki, Helsinki, Finland; 4 Research Center of Brain and Cognitive Neuroscience, Liaoning Normal University, Dalian, China

**Keywords:** Neuroscience, Psychology

## Abstract

Previous studies have associated visual working memory (VWM) capacity with the use of internal attention. Retrocues, which direct internal attention to a particular object or feature dimension, can improve VWM performance (i.e., retrocue benefit, RCB). However, so far, no study has investigated the relationship between VWM capacity and the magnitudes of RCBs obtained from object-based and dimension-based retrocues. The present study explored individual differences in the magnitudes of object- and dimension-based RCBs and their relationships with VWM capacity. Participants completed a VWM capacity measurement, an object-based cue task, and a dimension-based cue task. We confirmed that both object- and dimension-based retrocues could improve VWM performance. We also found a significant positive correlation between the magnitudes of object- and dimension-based RCB indexes, suggesting a partly overlapping mechanism between the use of object- and dimension-based retrocues. However, our results provided no evidence for a correlation between VWM capacity and the magnitudes of the object- or dimension-based RCBs. Although inadequate attention control is usually assumed to be associated with VWM capacity, the results suggest that the internal attention mechanism for using retrocues in VWM retention is independent of VWM capacity.

## Introduction

Visual working memory (VWM) is an essential mental storage system that can briefly store visual information, integrating information from external inputs to create dynamic and continuous experiences. After forming VWM representations, individuals can mentally access and control them, even if the visual input disappears^1,2^. Although VWM is flexible and goal-oriented, it can only retain limited information from the total sensory input. Using internal attention, which is the modulation and retention of internally generated information^3^, individuals can select or prioritize VWM content to facilitate efficient information storage. In the last two decades, many studies have examined internal attention’s influence on VWM^4,5^.

Internal attention’s effect on VWM representations has been extensively studied using retrocues^4^. In a typical VWM task with retrocues, participants are asked to remember a memory array and complete a subsequent test. After the memory array disappears, a retroactive cue (retrocue) is presented to indicate which memory item is the most likely to be tested. Researchers found that, in a valid retrocue condition (e.g., pointing to the location of the item to be tested), VWM performance was better than that in a neutral cue condition^6,7^; thus, participants can benefit from retrocues in a VWM task. Improved VWM performance through the utilization of retrocues is called a *retrocue benefit* (RCB). The acquisition of RCB indicates that internal attention can improve the retention of VWM information.

Previous studies of retrocues in VWM tasks have mainly used two types of retrocues: object-based and dimension-based. Object-based retrocues direct attention to one or several items in the memory array, and such retrocues can be further divided into endogenous cues (i.e., presented at a central location and directed toward *the target item*; e.g.^6,8–16^), exogenous cues (i.e., presented at the location of *the target item*; e.g.^13,17–21^), and feature cues (i.e., presented one particular feature of *the target item* to indicate the target object that participants must retrieve; e.g.^22–28^). Dimension-based retrocues, by contrast, direct attention toward *one visual dimension* (e.g., color or orientation) of all the memory items^29,30^. Notably, feature-based cues and dimension-based cues differ: feature-based cues are used to direct attention to a specific object (e.g., “the orientation of *the blue bar”*), while dimension-based cues are used to direct attention to a specific dimension of all objects instead of a specific object (e.g., “the orientation of *all bars*”).

Early studies mostly investigated object-based retrocues’ influence on VWM representations^4,6,7^. Only recently have researchers started to use retrocues in VWM tasks to investigate dimension-based internal attention^29–34^. This development is important because dimension-based internal attention may depend on mechanisms other than object-based internal attention. Object-based retrocues may enable individuals to reduce multiple VWM representations to one representation (the cued item), thus reducing memory load. When using dimension-based retrocues, individuals cannot reduce the total number of VWM representations, but they can reduce the amount of information needed to retain in each representation. Since the influencing mechanisms of object-based and dimension-based internal attention for retaining VWM information may be different, it is necessary to investigate the effect of internal attention on VWM using separate object- and dimension-based retrocues.

A variety of studies have demonstrated that people can obtain benefit from object-based retrocues in VWM tasks^4^. Recent studies have also demonstrated improvement in VWM performance facilitated by dimension-based retrocues^29,30,34^ (but see a study by Maniglia and Souza^33^ that found no evidence for the dimension-based RCB). So far, however, there has been little discussion about what factors affect the magnitudes of object-based and dimension-based RCBs.

The magnitudes of object-based and dimension-based RCBs may be associated with other cognitive abilities (e.g., VWM capacity). Many studies have found a correlation between individual differences in VWM capacity and attention control; for example, previous studies that used a change detection task to quantify VWM capacity found positive correlations between VWM capacity and the ability to filter distractors^35–37^. Our recent study also found that, when memory stimuli were presented for a sufficiently long period, participants’ VWM capacity positively correlated with their ability to voluntarily “trade off” VWM numbers and precision (*trade-off* here refers to individuals adjusting their VWM resource allocation to emphasize quantity or quality^38^). Since inadequate attention control is assumed to affect VWM capacity, inefficient use of object- or dimension-based internal attention to facilitate the retention of VWM information may also be associated with VWM capacity. However, no study has directly investigated the relationship between VWM capacity and the magnitudes of object-based and dimension-based RCBs.

The present study aimed to explore the relationships between VWM capacity and the magnitudes of object-based and dimension-based RCBs. We used a change detection task to quantify VWM capacity, and then asked participants to conduct VWM recall tasks with object- and dimension-based retrocues. We expected to replicate the findings of previous studies regarding object- or dimension-based RCBs^4,29,30,34^, which found significant object- and dimension-based RCBs at a population level (using the mean performance of the sample). Namely, we expected to find that VWM performance under a valid cue condition would be better than that under a neutral cue condition for both dimension-based and object-based cue tasks. More importantly, since previous studies have found that VWM capacity is positively associated with attention control^35–38^, a tentative hypothesis is that there are positive correlations between VWM capacity and the magnitudes of RCBs. Lastly, the mechanisms of object-based and dimension-based RCBs may differ, but the utilization of object- and dimension-based retrocues can still involve overlapping (but not necessarily equivalent) mechanisms of internal attention. We therefore predicted a positive correlation between the magnitudes of object-based and dimension-based RCBs.

## Method

### Participants

Using G ∗ power 3.1.9.2, we calculated the minimum sample size to gain sufficient statistical power to detect a correlation strength of at least *r* = 0.30, with a statistical power of (1 − *β*) = 0.80 and *α* = 0.05 significance level^39^. The results indicated that 67 participants were required.

Our study was conducted under the *Declaration of Helsinki* and approved by the Ethics Committee of Liaoning Normal University and the Ethics Committee of Sichuan Normal University. Sixty-eight volunteers (24 male and 44 female; mean age: 21.74 ± 2.155, range 18–28 years), who were college students studying at the aforementioned universities, participated in this study in return for compensation. All participants reported having normal or corrected-to-normal vision, normal color vision, and no history of neurological problems. Participants provided written informed consent before participating in the study.

### Materials and apparatus

The experiment comprised three different tasks. First, participants completed a color change detection task, with which we measured their VWM capacity. Next, they completed two recall tasks (one with an object-based retrocue, the other with a dimension-based retrocue). Half the participants completed the object-based task first, while the other half completed the dimension-based task first. The whole experiment was conducted in a dimly lit, soundproof room with 19-inch screens presenting the stimuli. The distance between participants and their screens was approximately 60 cm.

In the color change detection task, six colored squares, each 0.65° × 0.65° (visual degrees), were displayed in a memory array in a 9.8° × 7.3° region surrounding a fixed central dot. The distance between any two colors had to be at least 3.5° (center-to-center).

In the retrocue tasks, the stimuli were the same as the corresponding stimuli in our previous study^29^. The memory stimuli were three colored bars (each 1.1° in length, 0.4° in height), which were randomly presented at the four corners of an imaginary square with a diagonal of 6.0°. For both the dimension-based retrocue task and the object-based cue task, only endogenous cues were used, since the dimension-based cue task can only be applied with an endogenous cue. Specifically, in the dimension-based cue task, the dimension-based retrocues were the words “color and “orientation,” and the neutral retrocue was the word “random” (all words presented in Chinese). These retrocues were presented in black Simsun normal font (approximately 3.2° × 1.5°) at the center of the screen. In the object-based cue task, the object-based retrocue was a pair of central arrows (1.5° × 1.5°), both of which pointed to the location of one memory item. The neutral retrocue was a black cross (1.5° × 1.5°) at the center of the screen. For both object- and dimension-based cue tasks, the test array for the color recall trials comprised an outlined square and a color wheel (5.8° inner radius; 2.2° thickness), while that for the orientation task comprised an outlined square (1.2° × 1.2°) and a vertical white bar (1.1° × 0.4°). To reduce the similarity between memory items, the colors and orientations in the memory array were randomly selected from 360 possible colors (1–360, in one color step) and 180 possible angles (1–180, in one orientation degree), with any two bars separated by at least 30 orientation degrees and 60 color steps.

### Procedure

#### VWM capacity measurement

**Figure 1:**
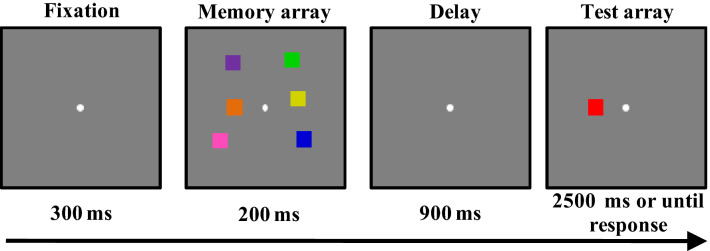
The task structure for the VWM capacity measurement.

The conventional single-probed color change detection paradigm was used for the VWM capacity measurement^37,38,40^. As Fig. 1 illustrates, each trial began with a fixed dot (300 ms), followed by a memory array of six colored squares (200 ms). Participants were instructed to remember these colored squares. After a delay (900 ms), a test array was presented with one colored square (2500 ms). The task was to indicate whether the color in test array was the same as that in a specific location within the memory array, stressing accuracy rather than response speed. All participants completed 100 trials of this task with a short break after the first 50 trials. Instructions at the beginning of this process informed participants about the task, and participants completed at least 16 practice trials before completing the main task. The VWM capacity measurement lasted approximately 10 min.

#### Dimension-based cue task

**Figure 2:**
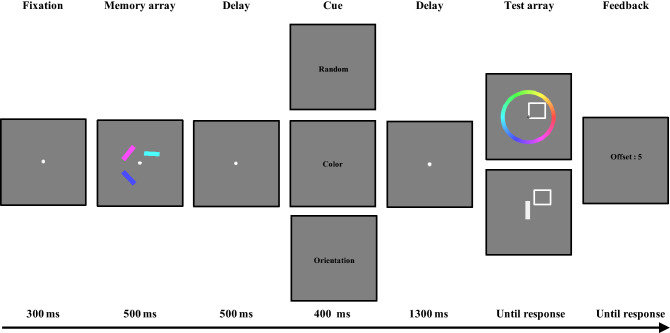
The task structure for the dimension-based cue task. The cue type in the upper row indicates the neutral retrocue, while the cue types in the middle and lower rows indicate the valid retrocues. For the test array, the upper row represents the color report trials, and the lower row represents the orientation report trials.

The procedure for the dimension-based cue task was the same as the corresponding procedure in our previous study^29^. Participants were asked to perform a dimension-based cue task with the trial structures depicted in Fig. 2. Dimension-based cue types (valid and neutral) and report types (color and orientation) were manipulated within-subject. Half of the trials included a valid dimension-based cue during the retention interval, and these valid cue trials were randomly dispersed among the trials that included a neutral cue. All trials began with a dot fixed for 300 ms at the center of the screen. A memory array containing three colored bars was then presented for 500 ms. Participants were instructed to memorize both the color and orientation of the bars. After 500 ms following the offset (response error in color steps or orientation degrees) of the memory array, a dimension-based cue (“color” or “orientation”; 100% valid) was presented at the center of the screen for 400 ms in the valid trials, while a neutral cue (“random”) appeared for 400 ms in the neutral trials. The cue was then followed by another retention interval, which lasted for 1300 ms. After the retention period, participants were asked to reproduce the color or orientation of one item in the memory array. The report type was selected at random for each trial. A white square outline appeared at the location of the VWM stimulus. If color was the report type, a color wheel was presented and participants were asked to report the memory item’s color in a white square outline; using a computer mouse, participants selected one of 360 color values. If orientation was the report type, an adjustable, vertical white bar was presented at a fixed point, and participants adjusted the white bar’s orientation to match the orientation of the tested item using a mouse. No time constraints were imposed on the participants. After the test array disappeared, feedback of the offset was provided. The next trial started 900–1100 ms after the feedback.

Forty-eight trials (the minimum trial number used in previous studies on dimension-based internal attention; e.g.^29,30,33^) were conducted for each condition (valid cue condition for color reports, neutral cue condition for color reports, valid cue condition for orientation reports, and neutral cue condition for orientation reports), with a total of 192 trials. Trials were fully randomized. The task lasted approximately 30 min. Instructions at the beginning of the task informed participants about the task, and participants completed at least 16 practice trials before completing the main task.

#### Object-based cue task

**Figure 3:**
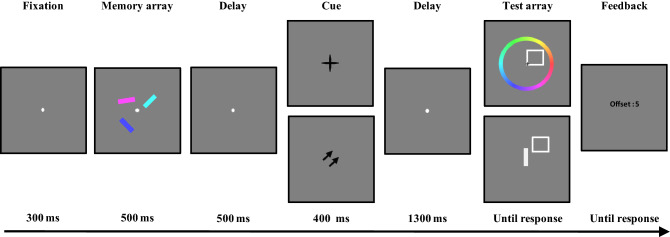
The task structure for the object-based cue task. The cue type in the upper row indicates the neutral retrocue, while the cue type in the lower row indicates the valid retrocue. For the test array, the upper row represents the color report trials, and the lower row represents the orientation report trials.

According to the trial structures depicted in Fig. 3, participants were asked to perform a recall task with object-based retrocues. Object-based cue types (valid and neutral) and report types (color and orientation) were manipulated within-subject. Participants needed to remember both the color and orientation of three memory items and then reproduce the color or orientation of one item in the memory array. The object-based cue task procedure resembled that of the dimension-based cue task, except regarding the retrocues. In the object-based cue task, the valid cue was an object-based retrocue (100% valid; a pair of central arrows pointing to the location of the item to be tested), and the neutral cue was a black cross presented at the center of the screen.

Like the dimension-based cue task, the object-based cue task comprised 48 trials for each condition (valid cue condition for color reports, neutral cue condition for color reports, valid cue condition for orientation reports, and neutral cue condition for orientation reports), with a total of 192 trials. The trials were fully randomized. The task lasted approximately 30 min. Instructions at the beginning of the task informed participants about the task, and participants completed at least 16 practice trials before completing the main task.

### Data analysis

#### VWM capacity measurement analysis

For the VWM capacity measurement, similar to studies that reported VWM capacity of participants^37,38,40,41^, we quantified each participant’s VWM capacity based on their results in the color change detection task. Since our study used the single-probe change detection task^42^, the standard formula proposed by Cowan^43^ was applied: *K* = *N* × (*H* − *F*), where *K* is the VWM capacity, *N* is the set size of the memory array (i.e., six in the present study), *H* is the hit rate or proportion of correct responses when a change is present, and *F* is the false alarm rate or proportion of incorrect responses when no change is present.

#### Offset analysis

For the object- and dimension-based cue tasks, we computed each participant’s response deviation for each experimental condition (valid cue condition for color reports, neutral cue condition for color reports, valid cue condition for orientation reports, and neutral cue condition for orientation reports) by subtracting the target item’s value from the response. One main dependent variable used to represent the recall task performance was the deviation’s absolute value, which we called the “offset”. A larger offset signified worse memory performance.

It is worth noting that the offset (VWM performance) depended on the defined color step or orientation degree. Because the response ranges of color (1–360 color steps) and orientation (1–180 orientation degree) differed, a larger offset in the color report trials than that in the orientation report trials did not mean that the color memory performance was worse than the orientation memory performance; thus, we did not directly compare the offset in the color report trials with the offset in the orientation report trials. Instead, we used the formula of the RCB index to standardize the magnitude of RCBs in the color and orientation report trials.

#### RCB index analysis

The other main dependent variable used to represent RCB magnitude was the RCB index, which we defined as:

$$RCB \,index= \frac{Offset\left(neutral\right)-Offset(valid)}{Offset(neutral)}.$$

“Offset(neutral)” and “offset(valid)” are the VWM performance index (i.e., offsets) under the neutral and valid cue conditions. The RCB index was therefore the percentage of relative improvement in the valid cue condition compared to the neutral cue condition; that is, the RCB index represented the magnitude of the benefit obtained from retrocues. Based on the above formula, we calculated the object- and dimension-based RCB indexes in the color report trials and orientation report trials.

#### Statistical analysis

To examine the existence of object- and dimension-based RCBs, we conducted repeated measures ANOVAs (analysis of variance) for offsets with different cue types (neutral cue vs. valid cue) and tasks (object-based cue task vs. dimension-based cue task) as within-subject factors. Two-tailed t-tests were conducted for follow-up comparisons of the object- and dimension-based cue tasks. Because the color and orientation reports in our recall tasks differed qualitatively, we conducted separate analyses for the offsets in the color and orientation report trials. To investigate the relationship between VWM capacity and the utilization of retrocues, we calculated the two-tailed Pearson’s correlation coefficients between VWM capacity and the RCB index (magnitude of RCB) for the different retrocues.

As in a previous study on VWM capacity^44^, we sorted participants into three groups on the basis of their VWM capacity (high capacity, medium capacity, or low capacity) to visualize the relationship between VWM capacity and RCB indexes. Moreover, to analyze the effect of VWM capacity on RCB indexes at the population level, we conducted repeated measures ANOVAs for the RCB index with reported features (color vs. orientation) and tasks (object-based cue task vs. dimension-based cue task) as within-subject factors, and capacity group (high capacity vs. medium capacity vs. low capacity) as a between-subject factor.

A significance level of *p* < 0.05 was used for all tests. All t-tests were conducted using a bootstrapping method (SPSS version 24.0; 1000 permutations with 95% confidence intervals), and Cohen’s d was used as an estimator of the effect size. Partial η^2^ was used as an estimator of the effect size for ANOVAs. A Bayes factor analysis (Bayesian t-test) was also used for reporting the t-test results^45^. The Bayes factor (BF_10_) provides an odds ratio for alternative/null hypotheses (values < 1 favor a null hypothesis and values > 1 favor an alternative hypothesis); for example, a BF_10_ of 0.5 would indicate that the null hypothesis is two times more likely than the alternative hypothesis.

## Results

### Offset results

**Figure 4:**
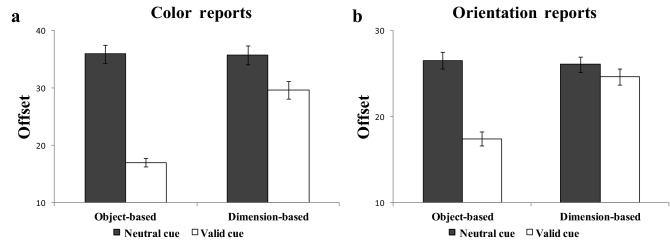
Offsets results for the neutral cue and valid cue conditions for each task, separated into (**a**) color report trials and (**b**) orientation report trials. “Object-based neutral cue” refers to the neutral cue condition in the object-based cue task. “Object-based valid cue” refers to the valid cue condition in the object-based cue task. “Dimension-based neutral cue” refers to the neutral cue condition in the dimension-based cue task. “Dimension-based valid cue” refers to the valid cue condition in the dimension-based cue task. Mean values with error bars show the standard errors of the means.

For the color report trials, Fig. 4a shows the offsets for each cue condition (neutral cue or valid cue) for each task (object-based cue task or dimension-based cue task). The ANOVA results showed the significant main effects of the cue conditions, F(1,67) = 165.404, p < 0.001, η^2^_p_ = 0.712, of the task, F(1,67) = 37.730, *p* < 0.001, η^2^_p_ = 0.360, and a significant interaction between cue conditions and tasks, F(1,67) = 69.336, *p* < 0.001, η^2^_p_ = 0.510. Follow-up comparisons showed that the offset under the valid cue condition was significantly lower than that under the neutral cue condition for both the object-based cue task, t(67) = 13.742, *p* < 0.001, CI_95%_ = [15.838, 21.864], d = 1.84, BF_10_ > 10,000, and the dimension-based cue task, t(67) = 5.606, *p* < 0.001, CI_95%_ = [4.071, 8.318], d = 0.46, BF_10_ > 10,000.

For the orientation report trials, Fig. 4b shows the offsets for each cue condition (neutral cue or valid cue) for each task (object-based cue task or dimension-based cue task). The ANOVA results showed significant main effects of the cue conditions, F(1,67) = 164.300, p < 0.001, η^2^_p_ = 0.710, of the task, F(1,67) = 47.859, *p* < 0.001, η^2^_p_ = 0.417, and a significant interaction between cue conditions and tasks, F(1,67) = 84.633, *p* < 0.001, η^2^_p_ = 0.558. Follow-up comparisons showed that the offset under the valid cue condition was significantly lower than that under the neutral cue condition for both the object-based cue task, t(67) = 14.640, *p* < 0.001, CI_95%_ = [7.907, 10.427], d = 1.24, BF_10_ > 10,000, and the dimension-based cue task, t(67) = 2.652, *p* = 0.01, CI_95%_ = [0.375, 2.539], d = 0.19, BF_10_ = 3.345. We conducted a correlation analysis to explore the relationship between VWM capacity and offset (memory performance) under each condition for different tasks. Because the focus of the study was on the relationship between VWM capacity and the RCB index, the results and discussion of the relationship between VWM capacity and memory performance are reported and discussed in the Supplementary Material 1.

These results of offsets showed that, for both the color and orientation report trials, participants were able to significantly improve their VWM performance using object- and dimension-based cues. These findings suggest that the object- and dimension-based RCBs in our study were significant.

### Correlations between individuals’ VWM capacity and the magnitudes of RCBs

**Figure 5:**
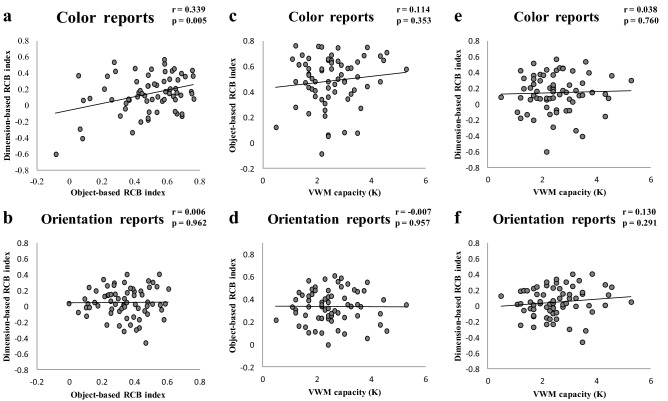
Scatter plots for the correlations between object-based RCB index, dimension-based RCB index, and VWM capacity. (**a**) Correlation across participants between object-based RCB index and dimension-based RCB index for color report trials. (**b**) Correlation across participants between object-based RCB index and dimension-based RCB index for orientation report trials. (**c**) Correlation across participants between VWM capacity (K) and RCB index for the color report trials for the object-based cue task. (**d**) Correlation across participants between VWM capacity and RCB index for the orientation report trials for the object-based cue task. (**e**) Correlation across participants between VWM capacity and RCB index for the color report trials for the dimension-based cue task. (**f**) Correlation across participants between VWM capacity and RCB index for the orientation report trials for the dimension-based cue task.

Table 1 shows the correlations and descriptive statistics for VWM capacity and the RCB indexes (magnitudes of RCBs) for object- and dimension-based tasks for the color report and orientation report trials (see Fig. 5c-f for scatter plots of the correlations). The skewness and kurtosis values were within acceptable ranges^46^. The correlation results showed no significant correlation between VWM capacity and the RCB indexes of the object- or dimension-based cue tasks (all r < 0.130, *p* > 0.291); that is, contrary to our expectations, we found no evidence for a correlation between VWM capacity and the magnitudes of RCBs.

**Table 1:** Descriptive statistics and correlations for VWM capacity and the RCB index for each condition.

Measure	1	2	3	4	5
1. VWM capacity	–				
2. RCB index for object-color reports	0.114	–			
3. RCB index for object-orientation reports	− 0.007	0.177	–		
4. RCB index for dimension-color reports	0.038	0.339**	0.235	–	
5. RCB index for dimension-orientation reports	0.130	0.054	0.006	0.049	–
Mean	2.52	48.62%	33.048%	14.29%	4.62%
*SD*	0.94	0.19	0.14	0.23	0.18
Skew	0.576	− 0.907	− 0.203	− 0.625	− 0.344
Kurtosis	0.322	0.435	− 0.519	0.625	− 0.022

“Object-color reports” refers to the color report trials in the object-based cue task. “Object-orientation reports” refers to the orientation report trials in the object-based cue task. “Dimension-color reports” refers to the color report trials in the dimension-based cue task. “Dimension-orientation reports” refers to the orientation report trials in the dimension-based cue task.

N = 68, **p < 0.01.

Interestingly, in the color report trials, the object-based RCB index had a significant positive correlation with the dimension-based RCB index (r = 0.339, *p* < 0.01) while no significant correlation was found between the magnitudes of the RCBs for the object- and dimension-based cues in the orientation report trials (r = 0.006, *p* = 0.962; Fig. 5a-b). It seemed that participants who effectively used object-based cues more effectively used dimension-based cues to improve their VWM performance in the color report trials. These results suggested that—at least for the color reports—the utilization of object-based retrocues was partly related to the utilization of dimension-based retrocues.

### RCB index results for different VWM capacity groups

The average VWM capacity estimate (K score) was 2.52 ± 0.94. Participants were sorted into three groups (high capacity, medium capacity, and low capacity) on the basis of their VWM capacity. The one-third split on the K scores resulted in 22 participants (9 male and 13 female) in the high VWM capacity group (M = 3.60 ± 0.64), 24 participants (10 male and 14 female) in the medium VWM capacity group (M = 2.42 ± 0.20), and 22 participants (5 male and 17 female) in the low VWM capacity group (M = 1.54 ± 0.36).

**Figure 6:**
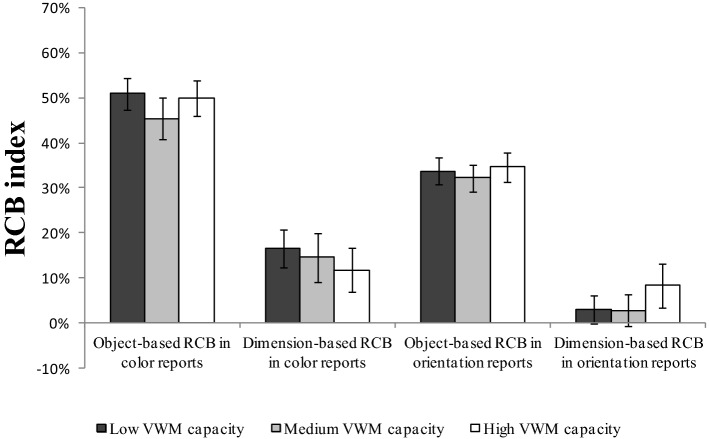
RCB indexes for the high VWM capacity, medium VWM capacity, and low-VWM-capacity groups, separated according to each reported feature in the different cue tasks. “Object-based RCB in color reports” refers to the RCB for the color report trials for the object-based cue task. “Dimension-based RCB in color reports” refers to the RCB for the color report trials for the dimension-based cue task. “Object-based RCB in orientation reports” refers to the RCB for the orientation report trials for the object-based cue task. “Dimension-based RCB in orientation reports” refers to the RCB for the orientation report trials for the dimension-based cue task. Mean values with error bars show the standard errors of the means.

Figure 6 shows the RCBs for each reported feature (color or orientation) for each task (dimension-based cue task or object-based cue task) for each capacity group (high VWM capacity, medium VWM capacity, or low VWM capacity). The ANOVA results showed significant main effects of the reported feature, F(1,67) = 28.662, *p* < 0.001, η^2^_p_ = 0.306, of the task, F(1,67) = 241.577, *p* < 0.001, η^2^_p_ = 0.788. All the other main effects and interaction effects were non-significant (all *p* > 0.192). These results showed no impact of VWM capacity on object- or dimension-based RCB index. Again, the results provided no evidence for the difference in the object- or dimension-based RCB index between the different VWM capacity groups.

## Discussion

The main purpose of the present study was to explore the relationship between VWM capacity and the magnitudes of RCBs. In line with previous findings supporting object- and dimension-based RCBs^4,29,30,34^, our results at the population level showed that VWM performance could be improved by using both object- and dimension-based retrocues. Our findings for the existence of RCBs at the population level enabled us to further investigate correlations at the individual level; however, we found no evidence for correlations between the magnitudes of RCBs and VWM capacity. Our results did not support the idea that the efficient use of internal attention to facilitate the retention of VWM information is associated with VWM capacity. Previous studies have associated participants’ VWM capacity with the ability of attention control to filter distractors or “trade off” quantity and quality of VWM representations^36–38,40^. The current results implied that, besides attention control, participants can use another kind of attention (internal attention) to affect VWM retention by using retrocues. Importantly, the mechanism for using retrocues to improve VWM differs from the mechanism for distractor filtering or trading off quantity and quality of VWM representations; thus, previous findings on the relationship between VWM capacity and attention control cannot explain the internal attention processing involved in the present study.

The magnitude of RCB may be affected by other memory factors besides VWM capacity. The interstimulus interval (ISI) between the memory array and retrocue was 500 ms in our study. It has been suggested that information can linger in iconic memory, which is a fast decaying store of visual information, for about 500 ms^47^. Participants who could retain information in the iconic memory for longer could have obtained greater RCB, because iconic memory assisted the allocation of internal attention; thus, the RCB indexes in our study may have been affected by individual differences in the duration of iconic memory retention. Using a longer ISI could prevent this potential problem. Moreover, in addition to iconic memory and VWM, recent studies have suggested that a third memory stage, termed fragile visual short-term memory (FM), exists between iconic memory and VWM. FM has a large capacity (more than eight items) and long duration^21,48–50^. FM can exist almost as long as VWM without interference from a new stimulus. Individuals can selectively transfer cued FM to VWM via internal attention to obtain RCB. Since the capacity of FM is independent of VWM capacity^21,48,49^, RCBs may be related to FM capacity rather than VWM capacity. Future research could examine the possible impacts of iconic memory and FM on RCBs.

Besides the relationship between VWM capacity and RCB indexes, we also examined the relationship between the RCB indexes for object-based retrocues and dimension-based retrocues. We found that in the color report trials, the participants who benefitted most from object-based retrocues also benefitted most from dimension-based retrocues, but there was no correlation between the object-based RCB and the dimension-based RCB in the orientation report trials. The results suggested that—at least for the color reports—the object-based and dimension-based RCBs may share an overlapping internal attention mechanism. A recently published study also examined the relationship between object-based and dimension-based RCBs^31^ and, in line with our results, found moderate positive correlations between object- and dimension-based RCB indexes. Previous studies have demonstrated that orientation stimuli require a larger VWM consolidation bandwidth than color stimuli^51,52^. Participants had more difficulty completing orientation report trials under valid cue conditions (resulting in a larger guess rate) for the dimension-based cue task than for the object-based cue task. This could have weakened the correlation between the object- and dimension-based RCB indexes in the orientation report trials, resulting in the only positive correlation in color report trials observed in our study.

It is worth noting that there was an inherent difference in the memory load imposed by the object-based and dimension-based cue tasks in our study. Although the neutral cue conditions for the object- and dimension-based cue task were similar, the task difficulty (storage requirements) for the valid cue conditions of the two tasks differed. In the object-based cue task, after the retrocue was presented, participants only needed to retain two features (one color and one orientation) of one object, but in the dimension-based cue task, they needed to retain three features (three colors or three orientations) of three objects. This led to worse performance under valid cue conditions for dimension-based cue tasks than for object-based cue tasks. As a result, the magnitude of dimension-based RCB was expected to be smaller than that of object-based RCB, and our results showed that the average RCB index for all participants obtained via object-based retrocues (41.05% ± 0.13) was larger than the average RCB index obtained via dimension-based retrocues (9.45% ± 0.15). This indicated that participants did not obtain as many benefits from dimension-based retrocues as from object-based retrocues because of our experimental design. The correlation between the magnitudes of the object- and dimension-based RCBs could have been weakened by the set size of the memory arrays in our study.

We should point out that the main purpose of our experimental design was to investigate the relationship between VWM capacity and the magnitude of RCBs, rather than directly compare object-based and dimension-based RCBs. We did not choose fewer items for the memory arrays (e.g., two double-feature items as in Hajonides et al.^31^’s study), because participants were expected to have near-perfect performance if they only needed to memorize two or fewer items^1^. The ceiling effect of VWM performance could have reduced the difference between the neutral cue condition and the valid cue condition for the retrocue task, resulting in a weakening of RCBs. Indeed, our previous study found that the dimension-based RCB index for remembering three double-feature items was larger than that for remembering two double-feature items^29^. Since the research on the dimension-based RCB is very limited, we designed the experiment to require participants to memorize three items instead of two items to maximize the dimension-based RCB.

It also seemed that the RCB indexes for the color report trials (48.62% ± 0.19 for the object-based cue task; 14.29% ± 0.23 for the dimension-based cue task) were larger than the RCB indexes for the orientation report trials (33.48% ± 0.14 for the object-based cue task; 4.62% ± 0.18 for the dimension-based cue task). Due to the qualitative difference between the offsets of the color (color steps) and orientation report trials (orientation degrees), the latter may not have been inherently more difficult than the former. This difference between the RCB indexes for the color and orientation report trials could have been affected by factors other than the features; for example, the set size of memory array could have affected the difference in RCB indexes between the color and orientation report trials. By using a similar paradigm for the dimension-based cue task, our previous results showed that when participants needed to memorize three items, the dimension-based RCB index for color report trials was 56% larger than for orientation report trials. However, when the participants only needed to memorize two items, the dimension-based RCB index for color report trials was only 3% larger than for the orientation report trials^29^; therefore, memorizing more items may have increased the difference in RCB indexes between the color report trials and orientation report trials. Future studies should systematically examine the effect of memory load on the relationship between different kinds of RCBs.

Although extensive studies over the past decade have repeatedly supported the acquisition of RCBs through using object-based retrocues^4^, only a handful of recent studies have investigated whether people can improve their VWM performance by using dimension-based retrocues^29–31,33,34^. The results of our study provide new evidence for the existence of dimension-based RCB. However, previous studies obtained contradictory results regarding dimension-based RCB. In addition to those studies supporting the existence of dimension-based RCB^29–31,34^, a recent study by Maniglia and Souza^33^ found no evidence for the existence of dimension-based RCB in a young adult group. Here, we propose two possible reasons for the null result in Maniglia and Souza^33^ study. Firstly, unlike object-based RCB, dimension-based RCB may be difficult to observe. In Maniglia and Souza^33^’s study, participants needed to remember six double-feature items in each trial That is, they still needed to retain six features after the dimension-based retrocues appeared. Since young adults’ VWM capacity is about three to four units^53^, the information that participants needed to retain exceeded their VWM capacity, which may cause the floor effect under both neutral cue and valid cue conditions, resulting in the null result for dimension-based RCB. Secondly, Maniglia and Souza^33^’s task was more complicated than other studies. Their trials using dimension-based retrocues were randomly dispersed among trials with dimension-based precues. Because the use of retrocues and precues involves different mechanisms, participants who are accustomed to using dimension-based precues may be less able to use dimension-based retrocues. Future studies on the dimension-based RCB should take these possibilities into account.

In conclusion, the current study provides new evidence for the existence of object- and dimension-based RCBs, suggesting that the influencing mechanisms of object- and dimension-based internal attention on VWM retention partly overlap. More importantly, although inefficient attention control is usually associated with VWM capacity^35–38,40^, we found no support for the claim that participants with high VWM capacity can obtain greater RCB than low-capacity participants using either object- or dimension-based retrocues; hence, individual differences in the RCB indexes cannot be explained by limited VWM capacity. Since far too little attention has been paid to the factors that affect the magnitudes of RCBs (especially the dimension-based), our study improves the understanding of RCBs as well as the internal attention mechanism in VWM retention.

## Supplementary Information

Supplementary Information.

## Data Availability

The datasets generated during and/or analysed during the current study are available from the corresponding author (lq780614@163.com, Qiang Liu) on reasonable request.
