# Supplementary material for: Individual differences in working memory capacity are unrelated to the magnitudes of retrocue benefits
Source: Sci Rep. 2021 Mar 31;11:7258. doi: 10.1038/s41598-021-86515-5 (PMC8012624; doi:10.1038/s41598-021-86515-5)
Supplement: Supplementary file 1 — Supplementary Information. [file 41598_2021_86515_MOESM1_ESM.docx]

**Supplementary Material**

**Individual Differences in Working Memory Capacity are Unrelated to the Magnitudes of Retrocue Benefits**

Chaoxiong Ye^1,2^, Qianru Xu^1,2^, Xinyang Liu^1^, Piia Astikainen^2^, Yongjie Zhu^3^, Zhonghua Hu^1^, Qiang Liu^1,4*^

1 Institute of Brain and Psychological Sciences, Sichuan Normal University, Chengdu, China

2 Department of Psychology, University of Jyvaskyla, Jyväskylä, Finland

3 Faculty of Information Technology, University of Jyvaskyla, Jyväskylä, Finland

4 Research Center of Brain and Cognitive Neuroscience, Liaoning Normal University, Dalian, China

*** Correspondence to**:

Qiang Liu, PhD

Institute of Brain and Psychological Sciences,

Sichuan Normal University, Chengdu, 610000, China

Email: lq780614@163.com

**The relationship between individuals’ VWM capacity and VWM performance in retrocue tasks**

To examine the relationship between VWM capacity and VWM performance (offsets) for recall tasks involving different retrocues, we calculated Pearson’s correlation coefficients (two-tailed) between VWM capacity (K values) and offsets (VWM performance) for each condition for the object- and dimension-based cue tasks.

Table 1 shows the correlations and descriptive statistics for VWM capacity and offsets (VWM performance) for each condition. As defined in the main text, the offset refers to the response error in degrees, which can represent VWM performance for recall tasks. It was calculated by the deviation’s absolute value; thus, a larger offset signified worse memory performance. The skewness and kurtosis values for each index were within acceptable ranges [^1^](#_ENREF_1). The correlations showed that VWM capacity significantly negatively correlated with each condition’s offset. Participants with a higher VWM capacity had a smaller offset (better performance) under each condition (neutral cue or valid cue) for both the object- and dimension-based cue tasks. These results suggested that participants with high VWM capacity performed better than participants with low VWM capacity on both object- and dimension-based cue tasks, regardless of the reported feature (color vs. orientation) and cue type (neutral vs. valid).

**Table 1.** ***Descriptive statistics and correlations for VWM capacity and the offsets for each condition.***

***Note:*** *N = 68. * = p < 0.05; ** = p < 0.01. “Neutral-object-color reports” refers to the color report trials under the neutral cue condition for the object-based cue task. “Valid-object-color reports” refers to the color report trials under the valid cue condition for the object-based cue task. “Neutral-object-orientation reports” refers to the orientation report trials under the neutral cue condition for the object-based cue task. “Valid-object-orientation reports” refers to the orientation report trials under the valid cue condition for the object-based cue task. “Neutral-dimension-color reports” refers to the color report trials under the neutral cue condition for the dimension-based cue task. “Valid-dimension-color reports” refers to the color report trials under the valid cue condition for the dimension-based cue task. “Neutral-dimension-orientation reports” refers to the orientation report trials under the neutral cue condition for the dimension-based cue task. “Valid-dimension-orientation reports” refers to the orientation report trials under the valid cue condition for the dimension-based cue task.*

As in a previous study on VWM capacity [^2^](#_ENREF_2), we sorted participants into three groups on the basis of their VWM capacity (high VWM capacity, medium VWM capacity, and low VWM capacity) to help visualize the relationship between VWM capacity and VWM performance (offsets). Moreover, to analyze the effect of VWM capacity on VWM performance at a population level, we conducted repeated measures ANOVAs for offsets for different cue conditions (neutral cue vs. valid cue), tasks (object-based cue task vs. dimension-based cue task), and capacity groups (high capacity vs. medium capacity vs. low capacity). For the color report trials, Figure 1 shows the offsets for each cue condition (neutral cue or valid cue) for each task (dimension-based cue task or object-based cue task) for each capacity group (high VWM capacity, medium VWM capacity, or low VWM capacity). The color report trials’ ANOVA results showed significant main effects for the cue condition, F(1,65) = 165.897, p < 0.001, η^2^_p_ = 0.718, the task, F(1,65) = 40.750, *p* < 0.001, η^2^_p_ = 0.385; and the capacity group, F(2,65) = 5.072, *p* = 0.009, η^2^_p_ = 0.135; a significant interaction between cue conditions and tasks, F(1,65) = 67.836, *p* < 0.001, η^2^_p_ = 0.511; and a significant interaction between cue conditions and capacity group, F(2,65) = 3.374, *p* < 0.040, η^2^_p_ = 0.094. All the other interaction effects were non-significant (all *p* > 0.299).

**Figure 1.** ***Offsets in different capacity groups for each condition for the color report trials.*** *“Object-based neutral cue” refers to the neutral cue condition for the object-based cue task. “Object-based valid cue” refers to the valid cue condition for the object-based cue task. “Dimension-based neutral cue” refers to the neutral cue condition for the dimension-based cue task. “Dimension-based valid cue” refers to the valid cue condition for the dimension-based cue task. Mean values with error bars show the standard errors of the means.*

For the orientation report, Figure 2 shows the offset for each cue condition (neutral cue or valid cue) for each task (dimension-based cue task or object-based cue task) for each capacity group (high VWM capacity, medium VWM capacity, or low VWM capacity). The orientation report trials’ ANOVA results showed significant main effects for the cue conditions, F(1,65) = 161.403, *p* < 0.001, η^2^_p_ = 0.713; the task, F (1,65) = 48.429, *p* < 0.001, η^2^_p_ = 0.427; and the capacity group, F(2,65) = 8.899, *p* < 0.001, η^2^_p_ = 0.215, and a significant interaction between cue conditions and tasks, F(1,65) = 86.460, *p* < 0.001, η^2^_p_ = 0.517. All the other interaction effects were non-significant (all *p* > 0.178).

**Figure 2.** ***Offsets in different capacity groups for each condition for the orientation report trials.*** *“Object-based neutral cue” refers to the neutral cue condition for the object-based cue task. “Object-based valid cue” refers to the valid cue condition for the object-based cue task. “Dimension-based neutral cue” refers to the neutral cue condition for the dimension-based cue task. “Dimension-based valid cue” refers to the valid cue condition for the dimension-based cue task. Mean values with error bars show the standard errors of the means.*

The results showed that participants’ VWM capacity affected their VWM performance (offset) in both the object- and dimension-based cue tasks. Importantly, the results showed a positive correlation between VWM capacity and VWM performance for each condition—regardless of whether they were for color or orientation report trials. Compared to participants with low VWM capacity, participants with high VWM capacity performed better under the neutral and valid cue conditions for both the dimension- and object-based cue tasks. Our findings were consistent with the results of [Robison and Unsworth ^3^](#_ENREF_3)'s study, which found that memory span (operation span, symmetry span, and reading span) can account for significant portions of performance variance (i.e., the accuracy of the change detection task) under precue, neutral, and retrocue conditions for VWM tasks.

It is worth noting that the change detection task (i.e., “VWM capacity measurement” in our study) and the recall task (i.e., retrocue tasks in our study) may not be independent. A study by [Schurgin, et al. ^4^](#_ENREF_4) showed that change detection and recall tasks measure the same property, which can be quantified as “visual memory strength”. Therefore, in our study, individual differences in VWM capacity—which were calculated using the hit rate and false alarm rate for the change detection task—reasonably related to the performance (offset) for recall tasks. Participants with high VWM capacity were more likely to store cued information (an item or feature) in their VWM, resulting in better performance in retrocue tasks. However, participants with low VWM capacity had a smaller chance to store cued information in their VWM, leading to more guess responses and worse performance in retrocue tasks. Therefore, it was reasonable to find correlations between VWM capacity and offsets for each condition in our study.

References

1 Brown, T. A. *Confirmatory factor analysis for applied research*. (Guilford publications, 2015).

2 Gaspar, J. M., Christie, G. J., Prime, D. J., Jolicoeur, P. & McDonald, J. J. Inability to suppress salient distractors predicts low visual working memory capacity. *Proceedings of the National Academy of Sciences of the United States of America* **113**, 3693-3698, doi:10.1073/pnas.1523471113 (2016).

3 Robison, M. K. & Unsworth, N. Variation in the use of cues to guide visual working memory. *Attention, perception & psychophysics* **79**, 1652-1665, doi:10.3758/s13414-017-1335-4 (2017).

4 Schurgin, M. W., Wixted, J. T. & Brady, T. F. Psychophysical scaling reveals a unified theory of visual memory strength. *Nature human behaviour* **4**, 1156-1172, doi:10.1038/s41562-020-00938-0 (2020).
